# Enhancement of Birefringence in Reduced Graphene Oxide Doped Liquid Crystal

**DOI:** 10.3390/nano10050842

**Published:** 2020-04-28

**Authors:** Mareddi Bharath Kumar, Mohammad Awwal Adeshina, Daekyung Kang, Youngho Jee, Taewan Kim, Muhan Choi, Jonghoo Park

**Affiliations:** 1 Department of Electrical Engineering, Kyungpook National University, Daegu 41566, Korea; 1992mbkr@gmail.com (M.B.K.); mohammadadeshina1@gmail.com (M.A.A.); kyoung_528@naver.com (D.K.); 2 Department Chemistry, Kyungpook National University, Daegu 41566, Korea; jyh@cresin.com; 3 Cresin Co., Ltd., Gyeongsangbuk-do 40040, Korea; 4 Department of Electrical Engineering and Smart Grid Research Center, Jeonbuk National University, Jeonju 54896, Korea; tw20027@gmail.com; 5 School of Electronics Engineering, Kyungpook National University, Daegu 41566, Korea; mhchoi@ee.knu.ac.kr

**Keywords:** reduced graphene oxide, liquid crystal, phase modulator, birefringence

## Abstract

We investigated the effect of reduced graphene oxide (rGO) doping on the birefringence of 5CB liquid crystal (LC). The characteristics of the synthesized rGO and LC-rGO composite with different rGO concentrations were analyzed by atomic force microscopy, X-ray photoelectron spectroscopy, white light polarized microscopy, voltage-dependent transmission measurement, and differential scanning calorimetry. We found that doping LC with an appropriate concentration of rGO enhances the birefringence of the LC. This is mainly due to the improved anisotropy of polarizability, which stems from the high shape anisotropy of rGO. However, the aggregation of rGO reduces the birefringence by decreasing the anisotropy of polarizability as well as the order parameter. Our study shows the promising potential of LC-rGO for developing various electro-optic devices that offer improved electro-optic effects.

## 1. Introduction

Doping liquid crystals (LCs) with nanoparticles (NPs) has attracted increasing attention in various research fields and industries because it not only changes the physical properties of LC but also provides additional functionalities. Various nanoparticles synthesized based on metals [1,2,3,4,5,6], dielectrics [7], semiconductors [8,9], carbon nanotubes [10,11,12], and ferroelectric [13,14] materials have been used as dopants to improve the electro-optic properties of the LC devices including a Fréedericksz transition voltage and a switching time. It has also been reported that ionic impurities could be co-doped with nanoparticles to improve the electro-optic performance of LC independent of temperature [15]. Among various NPs, the one that can increase dielectric anisotropy (Δ*ε*) without significantly reducing orientational order parameter has been considered as a preferred choice because it improves both a Fréedericksz transition voltage (*V_th_*) [16] and a switching time (*τ_on_*) [17] simultaneously through the following relationship:

(1) $$V_{th}=\sqrt{\frac{\pi^{2}K_{11}}{\varepsilon_{0}\Delta \varepsilon }}$$

(2) $$\tau_{on}\propto \frac{\gamma_{1}d^{2}}{\varepsilon_{0}\Delta \varepsilon \left(V^{2}-V_{th}^{2}\right)}$$

where *K*_11_ is the splay elastic constant, γ_1_ is the rotational viscosity, *d* is the thickness of the liquid crystal, and *V* is the applied voltage.

Recently, graphene [18,19,20,21,22] has been considered as a promising dopant for LC because of their unique physical properties and high shape anisotropic (i.e., high aspect ratio). The experimental results of the LC doped with pristine monolayer graphene flakes with an appropriate concentration exhibit giant enhancement in dielectric anisotropy [20,23,24]. It has also been reported that doping graphene oxide (GO) with an appropriate concentration decreases the threshold voltage [25]. According to the Maxwell-Wagner-O’Konski model [26,27], the high aspect ratio of the colloidal particle such as graphene and GO flakes yields the high anisotropy of the polarizability on which the dielectric anisotropy is strongly dependent. In addition, the molecular mean-field theory of a nematic liquid crystal doped with anisotropic nanoparticles predicts that the anisotropic nanoparticles promote the nematic ordering of the LC matrix, consequentially increasing the dielectric anisotropy [28]. Therefore, increasing dielectric anisotropy makes a significant contribution to enhancing the electro-optic properties of the LC, particularly the properties associated with the electric field-induced reorientation of n-director such as a Fréedericksz transition voltage and a switching time. There is another important parameter, birefringence, which should be carefully considered when choosing a dopant for an LC. The birefringence determines the optical path difference between the extraordinary and ordinary ray that passes through the LC, thus is a more relevant parameter for characterizing and designing liquid crystal devices, in particular for the devices that modulate the phase of the light using an electric field such as LC phase modulators [29,30,31,32] and LC lenses [33,34,35,36].

In general, graphene is a hydrophobic material, thereby it has poor solubility in solvents. Graphene with very low concentration will disperse in LC due to the insufficient graphene-graphene interactions. At higher concentrations the graphene-graphene interaction becomes significant and facilitates aggregation, resulting in decreased nematic ordering of the LC. Therefore, surface modification is required to disperse graphene in LC.

Here, we demonstrate the effect of chemically reduced graphene oxide (rGO) on the birefringence of the phase modulator fabricated using the homogeneously aligned LC doped with rGO (LC-rGO). Reduced graphene oxide is another form of graphene but it exhibits better dispersibility in solvents because of additional functional groups including carboxyl, epoxides and hydroxyl groups and is easier to mass-produce [37]. We found that the doping rGO in LC with an appropriate concentration enhances the birefringence of LC, consequentially, increases the total phase retardation of the phase modulator. The enhancement of the birefringence was mainly due to the improved polarizability that compensates for the decrease in the order parameter caused by rGO doping.

## 2. Design and Fabrication

**Figure 1:**
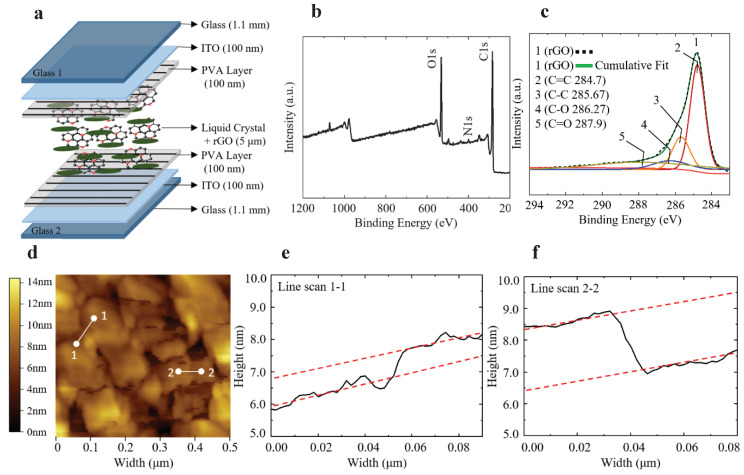
(**a**) Structure of the LC-rGO phase modulator. (**b**) XPS Survey of rGO and (**c**) high-resolution XPS spectra for C1s. (**d**) AFM image of rGO. (**e**) Line scan of Section 1-1 showing rGO thickness of 1.06 nm (2 layers). (**f**) Line scan of Section 2-2 showing rGO thickness of 1.675 nm (3 layers).

Figure 1a shows the structure of the phase modulator implemented by the rGO doped 5CB LC (LC-rGO). The fabrication of the phase modulator was started from cleaning indium tin oxide (ITO) coated glass substrates (Sigma Aldrich, St. Louis, MO, USA) with acetone, isopropyl alcohol (IPA), and deionized (DI) water in a sonication bath for 5 min, respectively. The thicknesses of ITO and the glass slide were 100 nm and 1.1 mm, respectively. The substrates were then exposed to UV ozone for 1000 s to make the surface hydrophilic. Polyvinyl alcohol (PVA, 2 wt%) was dissolved in DI water. The solution was stirred and heated at 90 ℃ for 2 h. The PVA solution was spin-coated on the glass substrate at the speed of 3000 rpm for 30 s and then baked for 1 h at 90 ℃ on a hot plate. The PVA layers on glass substrates were rubbed with a velvet cloth in an antiparallel direction. Two glass substrates were bonded using double-sided adhesive films (Nitto, Umeda, OSA, Japan) with a thickness of 5 µm, a width of 5 mm, and a length of 35 mm placed on the rims of one substrate. The area where the double-sided adhesive films were placed, was masked using kapton tape (DuPont, Wilmington, DE, USA) during the spin coating of PVA solution. Pressure was applied to the two substrates after assembling and before filling the LC. The LC with different weight concentrations of rGO was prepared by vortex-mixing rGO in a 5CB LC for 60 s, followed by sonicating for 2 h and heating at 90 ℃ for 30 min. At this temperature, the 5CB LC became isotropic and it allowed for better dispersion of rGO. The LC-rGO was filled between two substrates by capillary action at 60 ℃.

The rGO was prepared by reducing GO produced by the modified hummers method from the graphite powder. A mixture of 2 g of graphite having a mean size of 200 mesh and purity of 99%, 2 g of sodium nitrate (NaNO_3_), and 100 mL of sulfuric acid (H_2_SO_4_) with 95 wt% was prepared and stirred for 1 h. After cooling down the mixture in an ice bath, 10 g of potassium permanganate (KMnO_4_) was added and magnetically stirred until the solution became brown. Three hundred milliliter of deionized water was added to the solution slowly during the stirring. To remove excess KMnO_4_, 10 mL of hydrogen peroxide (H_2_O_2_) was added and stirred. Deionized (DI) water was then added and centrifuged. The supernatant was removed and residuals were washed using DI water and centrifuged five times. The GO solution was dried in the oven at 80 ℃ for 3 h to obtain the GO powder. One gram of GO were dissolved in 400 mL of DI water during sonication. After heating the GO solution up to 100 ℃, 10 mL of hydrazine was added and heated for 1 h. The resulting rGO was then washed using DI water and dried in the oven at 80 ℃ for 3 h. One gram of rGO was then dissolved in 100 mL of isopropyl alcohol and mixed using a rotating mixer for 10 min followed by the solution ball milling for 2 h. Figure 1b presents the survey X-ray photoelectron spectroscopy (XPS) spectrum of rGO, showing the N1s peak in addition to the C1s and O1s peaks. The N1s peak is attributed to the nitrogen doping by hydrazine reduction [38]. Figure 1c shows the C1s XPS spectrum which can be divided into four different peaks centered at 284.70, 285.67, 286.27, and 287.90 eV, corresponding to the C=C, C–C, C–O, and C=O groups, respectively. Figure 1d shows the atomic force microscopy image of the rGO. Figure 1e,f shows the thicknesses of the rGO along the line section 1-1 and 2-2, respectively. The thicknesses were measured to be 1.060 nm and 1.675 nm. Since the thickness of a chemically reduced monolayer GO sheet is 0.5–0.7 nm, the measured thicknesses correspond to two and three layers, respectively. [39,40,41]

## 3. Results and Discussions

**Figure 2:**
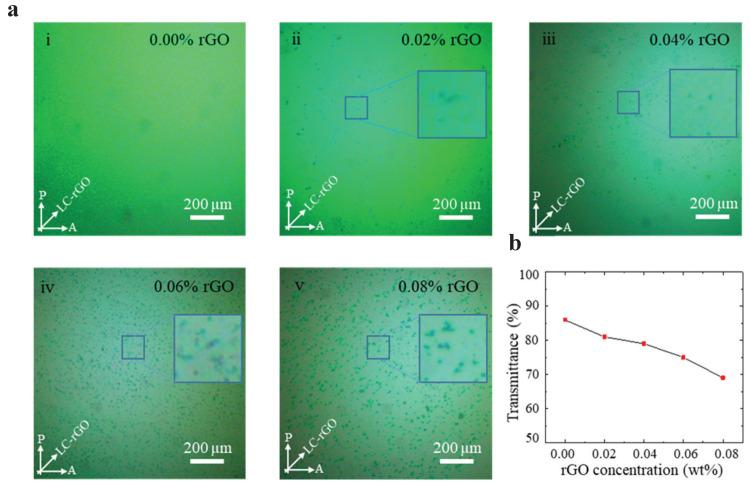
(**a**) Polarized optical microscope images of the pure LC (**i**) and LC-rGO with different concentrations rGO (**ii**–**v**). Insets are the magnified view of the area in the vicinity of rGO aggregates and show perturbation of nematic order around the rGO aggregates for the concentrations of 0.04 to 0.08 wt%. (**b**) Optical transmittance as a function of rGO concentrations, which decreases as rGO concentration increases.

The textures for the pure LC and LC-rGO with different rGO concentrations were analyzed by the white light polarized optical microscope (POM) as shown in Figure 2a. For the concentration of 0.02 wt%, the POM image shows the area with a uniform color and dark spots, corresponding to the uniform nematic texture and rGO aggregates, respectively. The uniform nematic texture in the POM image indicates the spontaneous alignment of rGO flakes in parallel to the far-field n-director of LC by the π-π electron stacking between the hexagonal lattices in rGO and benzene rings in the LC [42,43]. The uniform nematic texture also implies that the edges of the rGO flakes do not perturb the n-director of LC unless the rGO flakes agglomerate. The number and size of rGO aggregates increases as the rGO doping concentration increases and the perturbation of the n-director of LC around the rGO aggregates was observed for high rGO concentrations as shown in the inset of Figure 2a (iii–v). Since the rGO aggregates worsen the orientational order of the LC, one of the factors that determine the birefringence of the LC, it is critical to inhibit the aggregation of rGO flakes in order to take advantage of rGO doping. The aggregation of rGO flakes can be further minimized by adding nanosurfactants in the rGO-LC [37].

Figure 2b shows the optical transmittance of the LC-rGO with different rGO concentrations. The optical transmittance was measured as the ratio of the intensity of the incident light to the amount of the light that passes through the LC-rGO phase modulator using the power meter (Newport 1936-r) and a He-Ne laser at the wavelength of 543 nm. The intensity of the incident light was set to the same power for all samples. The polarization direction of the incident light was aligned in parallel to the optical axis of the LC-rGO phase modulator. Since the absorption of light in rGO increases with increasing rGO concentration, the transmittance decreased as the rGO concentration increased.

**Figure 3:**
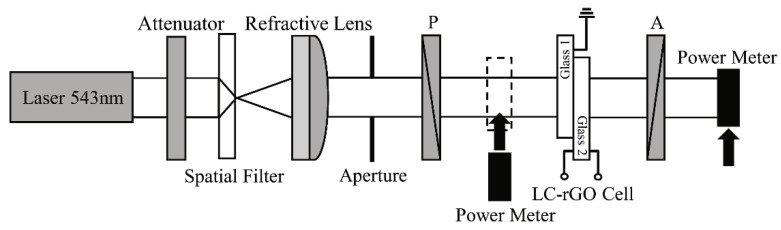
Voltage-dependent optical transmission measurement setup for the LC-rGO phase modulator.

**Figure 4:**
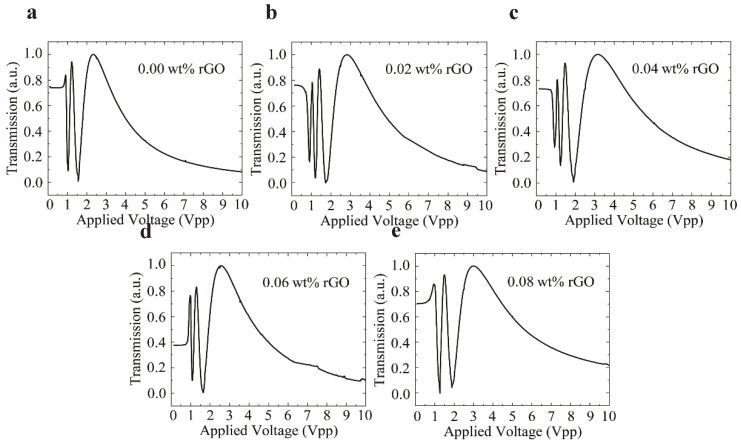
The voltage-dependent optical transmission for LC-rGO with different rGO concentrations (**a**–**e**). The LC-rGO with a doping concentration of 0.02 (**b**), and 0.04 wt% (**c**) shows the higher number of oscillations, indicating increased birefringence.

**Figure 5:**
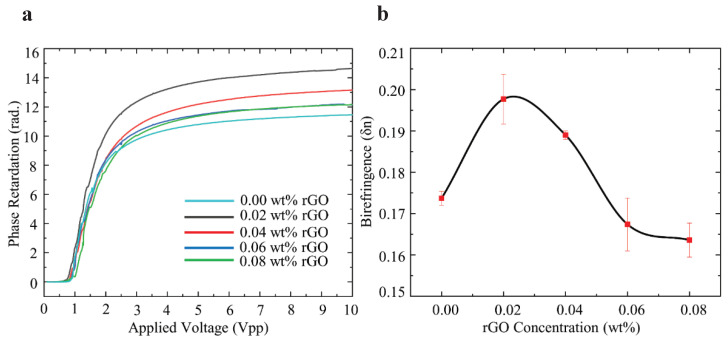
(**a**) Relative phase retardation as a function of the voltage for the phase modulator implemented by the pure LC and LC-rGO with different concentrations. (**b**) The birefringence of the LC-rGO phase modulator as a function of rGO concentrations, showing enhanced birefringence.

To quantify the changes in the birefringence by rGO doping, the voltage-dependent optical transmission measurement was performed. Figure 3 shows the measurement setup for the voltage-dependent optical transmission of the homogeneously aligned LC-rGO between glass substrates. The setup comprised a He-Ne laser with a wavelength of 543 nm, an attenuator, a spatial filter, a refractive lens, an aperture, a polarizer and the LC-rGO cell, an analyzer, and a power meter. The measurements were performed in crossed polarizers, and the polarizer azimuth angle (polarization direction) was 45° with respect to the optical axis of the LC-rGO phase modulator. The bipolar square wave pulse with a frequency of 1 kHz and zero DC bias was applied for the measurement. Figure 4a–e shows the voltage-dependent optical transmission of LC-rGO phase modulator with different rGO concentrations. The optical transmission oscillates between the minimum and maximum value as a function of voltage. The voltage-dependent optical transmissions for LC-rGO with rGO concentration of 0.02 and 0.04 wt% exhibit a higher number of oscillations than that of intrinsic LC, indicating increased effective birefringence. Figure 5a shows the voltage-dependent phase retardation of the LC-rGO phase modulator. It should be noted that the phase retardation is strongly dependent on the thickness and birefringence of the LC-rGO. Therefore, the thickness variation among cells should be carefully considered. Figure 5b shows the effective birefringence of the LC-rGO extracted from the phase retardation (Figure 5a) and the thicknesses of the LC-rGO layers. The thicknesses of four different positions of each empty LC-rGO cell were measured using a UV-Visible spectrometer and averaged. The average thickness (standard deviation) of an LC-rGO layer with concentrations of 0.00, 0.02, 0.04, 0.06 and 0.08 wt% were 5.704 (±0.055), 6.402 (±0.268), 6.041 (±0.016), 6.284 (±0.233), and 6.430 μm (±0.156), respectively. The average effective birefringence (standard deviation) of the LC-rGO with a concentration of 0.00, 0.02, 0.04, 0.06 and 0.08 wt% were 0.174 (±0.002), 0.198 (±0.006), 0.189 (±0.001), 0.167 (±0.006), and 0.164 (±0.004), respectively. The birefringence of the intrinsic LC is in good agreement with the value provided by the supplier. The birefringence of LC-rGO exhibits a higher value than that of the intrinsic LC in the range of 0.02 to 0.04 wt% rGO concentrations, and a further increase in doping concentration lowers the birefringence.

The birefringence of a nematic LC is determined by the order parameter and anisotropy of polarizability of the individual molecules. The relationship between the order parameter (S), the anisotropy of polarizability (Δα), and birefringence ($\Delta n=n_{e}-n_{o})\;$can be described by following the Vuks expression [44]:

(3) $$\frac{S\Delta \alpha }{\bar{\alpha }}=\frac{{n_{e}}^{2}-{n_{o}}^{2}}{{\bar{n}}^{2}-1}$$

where $n_{e}$ and $n_{o}$ are the extraordinary and ordinary refractive index, $\bar{\alpha }=1/3\left(\alpha_{x}+\alpha_{y}+\alpha_{z}\right)$, and $\bar{n}=1/3\left(n_{x}+n_{y}+n_{z}\right)$.

**Figure 6:**
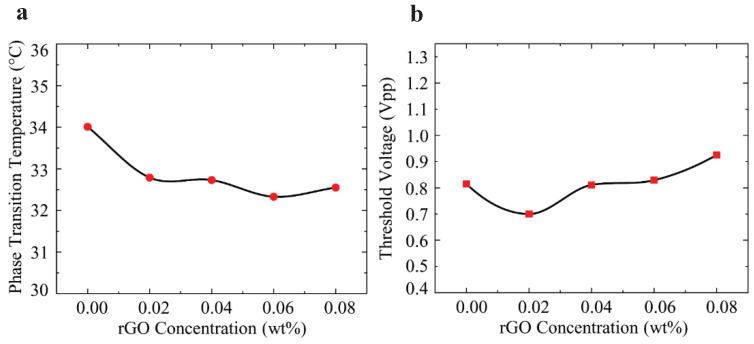
(**a**) Nematic to isotropic transition temperature as a function of rGO concentration and (**b**) measured threshold voltage.

To investigate the effect of rGO doping on the order parameter, the nematic to isotropic phase transition temperature (*T_NI_*) of the LC-rGO was measured using a differential scanning calorimeter as shown in Figure 6a. This shows that the addition of rGO decreases *T_NI_* for all doping concentrations. The relationship between *T_NI_* and the order parameter (S) can be described by following Haller’s empirical expression [45]:

(4) $$S={\left(1-\frac{T}{T_{NI}}\right)}^{\beta }$$

where *T* is the temperature at which the measurement is made, and *β* is a unitless fitting parameter. Therefore, the decrease in *T_NI_* is attributed to the reduced-order parameter for all rGO concentrations. This result and Equation (3) imply that increased birefringence for the rGO concentration of 0.02 and 0.04 wt% is mainly due to the increased anisotropy of polarizability which compensates for the decreases in the order parameter. When subjected to an alternating electric field, rGO is polarized and both rGO and LC gain dipole moments. The torque exerted on rGO not only rotates the rGO itself but also facilitates the rotation of LC anchoring on both sides of the rGO surface. Thus, the increased anisotropy of polarizability of the LC-rGO results in increased birefringence of the LC-rGO composite. For higher doping concentrations, the aggregation of rGO lowers birefringence by decreasing both the order parameter and the anisotropy of polarizability. Figure 6b shows the threshold voltage as a function of doping concentrations. For the concentrations of 0.02 and 0.04 wt%, the threshold voltage decreased compared to that of the intrinsic LC, whereas for higher doping concentrations, the threshold voltage increased. The threshold voltage is determined by the dielectric anisotropy (Δε) and the splay elastic constant (*K_11_*) as shown in Equation (1). To investigate the effect of the order parameter and polarizability anisotropy on the threshold voltage, we described dielectric anisotropy and the splay elastic constant in terms of order parameter and anisotropy of polarizability by [46,47,48]:

(5) $$\Delta \varepsilon =\mathrm{NhF}\left\{\Delta \alpha -\left(F\mu^{2}/2kT\right)\left(1-3cos^{2}\left(\beta \right)\right)\right\}S$$

(6) $$K_{11}=C_{11}V_{n}^{-7/3}S^{2}$$

where *N* is the number density, *h* is the cavity field factor, *F* is the Onsager reaction field, Δα is the polarizability anisotropy, μ is the dipole moment, *kT* is the thermal energy, β is the angle between the dipole moment and the primary molecular axis. *C_11_* is the reduced splay elastic constant, and *V_n_* is the mole volume. Therefore, the threshold voltage can be described by the order parameter and dielectric anisotropy as follows:

(7) $$V_{th}=\sqrt{\frac{C_{11}V_{n}^{-7/3}S}{\varepsilon_{0}NhF\left(\Delta \alpha -\gamma \right)}}$$

where $\gamma =\left(F\mu^{2}/kT\right)\left(1-3cos^{2}\left(\beta \right)\right)$. The threshold voltage is proportional to the square root of the order parameter and is inversely proportional to the square root of the polarizability anisotropy. The decrease in threshold voltage for the doping concentration of 0.02 and 0.04 wt% is attributed to the decrease in the order parameter and increase in polarizability anisotropy. For higher concentrations, it can be concluded that both the order parameter and anisotropy of polarizability decrease, however, the decrease in anisotropy of polarizability outweighs the decrease in the order parameter, thus, resulting in an increased threshold voltage.

## 4. Conclusions

We have demonstrated that the addition of an appropriate concentration of rGO enhances the birefringence of LC. It has been found that the enhancement of birefringence is mainly due to the increased anisotropy of polarizability, which compensates for the decrease in the order parameter. The high shape anisotropy of rGO provides improved anisotropy of polarizability. However, the aggregation of rGO reduces the birefringence by decreasing the anisotropy of polarizability as well as order parameter. Our study suggests that the LC doped with rGO can be potentially applied to a variety of electro-optic devices for an improved electro-optic effect and reduced device thickness.
